# Room-Temperature Solid-State UV Cross-Linkable Vitrimer-like Polymers for Additive Manufacturing

**DOI:** 10.3390/polym14112203

**Published:** 2022-05-29

**Authors:** Jian Chen, Ya Wen, Lingyi Zeng, Xinchun Wang, Hongmei Chen, Wei Min Huang, Yuefeng Bai, Wenhao Yu, Keqing Zhao, Ping Hu

**Affiliations:** 1 College of Chemistry and Materials Science, Sichuan Normal University, Chengdu 610066, China; jianchen202205@163.com (J.C.); wenya_10030420@163.com (Y.W.); zenglingyi307@163.com (L.Z.); puremilk2801860969@163.com (X.W.); byf610327@163.com (Y.B.); yuwenhao@sicnu.edu.cn (W.Y.); kqzhao@sicnu.edu.cn (K.Z.); 2 School of Mechanical and Aerospace Engineering, Nanyang Technological University, Singapore 639798, Singapore

**Keywords:** vitrimer, shape memory effect, cross-linking, additive manufacturing

## Abstract

In this paper, a UV cross-linkable vitrimer-like polymer, ureidopyrimidinone functionalized telechelic polybutadiene, is reported. It is synthesized in two steps. First, 2(6-isocyanatohexylaminocarbonylamino)-6-methyl-4[1H]-pyrimidinone (UPy-NCO) reacts with hydroxy-functionalized polybutadiene (HTPB) to obtain UPy-HTPB-UPy, and then the resulted UPy-HTPB-UPy is cross-linked under 365 nm UV light (photo-initiator: bimethoxy-2-phenylacetophenone, DMPA). Further investigation reveals that the density of cross-linking and mechanical properties of the resulting polymers can be tailored via varying the amount of photo-initiator and UV exposure time. Before UV cross-linking, UPy-HTPB-UPy is found to be vitrimer-like due to the quadruple hydrogen-bonding interactions. The UPy groups at the end of the chain also enable for rapid solidification upon the evaporation of the solvent. The unsaturated double bonds in the HTPB chains enable UPy-HTPB-UPy to be UV cross-linkable in the solid state at room temperature. After cross-linking, the polymers have good shape memory effect (SME). Here, we demonstrate that this type of polymer can have many potential applications in additive manufacturing. In the cases of fused deposition modelling (FDM) and direct ink writing (DIW), not only the strength of the interlayer bonding but also the strength of the polymer itself can be enhanced via UV exposure (from thermoplastic to thermoset) either during printing or after printing. The SME after cross-linking further helps to achieve rapid volumetric additive manufacturing anytime and anywhere.

## 1. Introduction

A range of technologies have been developed for additive manufacturing (AM) [1]. Fused deposition modelling (FDM) and direct ink writing (DIW) are two typical ones. High interlayer bonding is required in both of them for high structural strength. Volumetric additive manufacturing (VAM), which selectively solidifies a part of photosensitive liquid within a contained volume, is relatively new, but has the potential for rapid AM [2]. However, same as most existing technologies, it is not applicable for AM at anytime and anywhere. In microgravity environments (e.g., in space missions) and harsh environments (e.g., on ships and vehicles during maneuvering, or in airplanes during flight), AM with liquids or powders is highly problematic.

The concept of rapid solid-state volumetric additive manufacturing, proposed by Wang et al. [3], aims for rapid AM in any environment. Different from current VAM [2], UV cross-linking in solid-state VAM is carried out on a solid piece of polymer. This concept has been realized using a UV cross-linkable thermal gel [4], which is solid at room temperature, and becomes an easy-to-flow liquid upon cooling to below 10 °C. Thus, after cross-linking at room temperature in the solid state, the un-cross-linked part can be removed by washing in iced water. The shape memory effect refers to the capability of a material to return its original shape after being severely and quasi-plastically deformed, but only at the presence of the right stimulus. This feature is applicable to most polymeric materials, including most thermset [5], and ensures high dimensional accuracy after the un-cross-linked part is removed. As for high-strength polymers, UV cross-linkable vitrimer, which has a reversible/dynamic cross-linking network so that it is thermoset at lower temperatures and becomes thermoplastic at higher temperatures, should be the right candidate for rapid solid-state VAM [6].

The hydrogen bond resulted by ureidopyrimidinone (UPy) is dynamic. Hence, a polymer with UPy groups has interesting functions, such as the shape memory effect and self-healing [7,8,9,10]. Due to the strong quadruple hydrogen bonding of UPy, oligomers can be assembled into supramolecular polymers, which are ductile [11,12]. The formation and breakage of the quadruple hydrogen bonds are affected by the temperature and right solvent [13,14]. Hence, such kinds of polymers are under the category of vitrimer or vitrmer-like [15], and can be used in the same way as normal thermoplastics in 3D printing via, for instance, FDM and DIW. Hayes et al. have successfully applied this kind of biocompatible supramolecular polymer as the “ink” for 3D inkjet printing [16].

Telechelic polybutadiene (HTPB) has an unsaturated double bond, which can undergo radical reaction, resulting in cross-linking. UPy-functionalized HTPB, as a special type of room-temperature solid-state cross-linkable vitrimer, can be used in 3D printing not only for enhanced bonding between layers in, for instance, FDM and DIW during or after printing, but also for rapid solid-state VAM in any environment.

**Figure 1:**
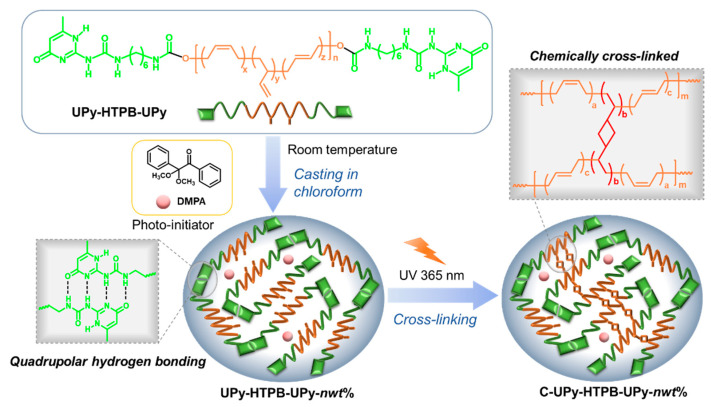
Material preparation procedure (*n* represents the mass fraction of DMPA).

In this study, a series of UPy-functionalized telechelic HTPB samples are prepared. As shown in Figure 1, 2(6-isocyanatohexylaminocarbonylamino)-6-methyl-4[1H]-pyrimidinone (UPy-NCO) is connected with hydroxy-functionalized polybutadiene (HTPB) to obtain UPy-HTPB-UPy. The polybutadiene is composed of cis and trans 1,4 olefins and 1,2 olefins [7]. After solvent evaporation, solid UPy-HTPB-UPy is formed due to the quadrupolar hydrogen-bonding interaction of UPy unites [17]. If a photo-initiator (e.g., bimethoxy-2-phenylacetophenone, DMPA) is added, under 365 nm UV light, UPy-HTPB-UPy can be cross-linked. The amount of DMPA and UV exposure time can be controlled to tailor the density of cross-linking [18]. Consequently, there are two types of cross-linking in the resulted polymers; one is permanent due to photo cross-linking and the other is dynamic as the result of the quadrupolar hydrogen-bonding interaction.

## 2. Materials and Experimental

### 2.1. Materials and Synthesis

*Materials*: Polybutadiene (HTPB, Mn = 4200 g/mol), 2-amino-4-hydroxy-6-methylpyrimidine (MIS), hexamethylene diisocyanate (HDI), and dibutyltin dilaurate (DBTDL) were purchased from Sarn Chemical Technology (Shanghai) Co., Ltd., China. Benzoin dimethyl ether (DMPA), dichloromethane, chloroform, methanol, and silica gel (for column chromatography) were purchased from Chengdu Kelong Chemical Reagent Factory, China. All reagents were used as received.

*Synthesis of Isocyanato-Terminated Pyrimidone (UPy-NCO)*: MIS (4.48 g) and HDI (40 g) were added into a 100 mL three-necked flask and stirred at 100 °C for 24 h protected by N_2_. After air cooled to room temperature (approximately 20 °C) and filtered, the resulting white solid piece was washed 8–9 times with petroleum ether (approximately 40 mL was used each time). A total of 9.85 g of product was obtained after vacuum drying at 50 °C for 24 h (yield 94%).

*Synthesis of UPy-HTPB-Upy*: Following that reported in [17], Upy-NCO (0.781 g), HTPB (3 g) and 50 mL chloroform were added into a 100 mL three-necked flask, and then stirred evenly with two drops of DBTDL added in. The reaction was protected by N_2_ at 60 °C for 16 h. Then, 5 g of 200–300 mesh silica gel was added in. After 16 h of reaction, 200 mL of CH_2_Cl_2_ was added in for dilution, and then the silica gel was removed by suction filtration to obtain viscous colorless transparent liquid. After most of the solvent was removed by rotary evaporation, a bit methanol was added until a milky white viscous precipitate appeared. It was then dissolved in a small amount of chloroform and then precipitated in methanol. This process was repeated twice. Vacuum drying at 50 °C for 24 h resulted in 3.12 g of transparent elastomer with a yield of 91.2%.

*Synthesis of C-UPy-HTPB-UPy*: Here, refer to Scheme S1 in the supplementary material. After dissolving 0.5 g of the elastomer in CH_2_Cl_2_, DMPA (photo-initiator, 2 wt%, 3 wt%, 4 wt%, or 5 wt%) was added in. After even stirring and then pouring into a polytetrafluoroethylene mold, a solid piece was obtained. Cross-linked film (thickness: 1.5 mm) was obtained via irradiation under a 25 W, 365 nm ultraviolet light. The degree of cross-linking was tailored by varying the exposure time.

### 2.2. Characterization

A Fourier transform infrared spectroscopy (FTIR) test was performed on a Thermo Electron Nicolet 570 spectrometer using film samples at room temperature in attenuated total reflectance (ATR, with SeZn) mode in the range of 600–4000 cm^−1^ with 64 scans and a resolution of 4 cm^−1^. For the variable temperature FTIR, the sample was prepared by dropping the solution onto a KBr pill and was tested in transmission mode in the range of 400–4000 cm^−1^ with 64 scans and a resolution of 4 cm^−1^.

A proton nuclear magnetic resonance (^1^H NMR) spectroscopy test was carried out on a Varian 400 MHz spectrometer at 399.98 MHz in CDCl_3_ (tetramethylsilane as an internal standard).

A differential scanning calorimetry (DSC) test was performed using a DSC Discovery (TA Instruments, New Castle, DE, USA) from −50 to 150 °C at a ramp rate of 10 °C min^−^^1^ under a nitrogen stream of 25 mL min^−1^.

Dynamic mechanical analysis (DMA) was carried out on a DMA Q850 (TA Instruments) in film tension mode (stress control). The sample (1.5 mm thick) was cut into the required length and width of 30 mm× 4 mm. The storage modulus (tensile modulus, E′) was obtained under the amplitude control mode upon heating from −90 to 150 °C at a heating rate of 3 °C min^−1^ and frequency of 1 Hz.

DMA was also used to characterize the shape memory performance of the cross-linked samples. Again, the test was in film tension mode (stress control), the applied heating/cooling speed was 3 °C/min, and the loading speed was 50 KPa/min. The detailed procedure of testing was as follows:

(i) The original strain is ε_1_ (0 in the first cycle). Heat the sample to 90 °C (the temperature that quadruple hydrogen bonding breaks) and hold for two minutes. After loading, the corresponding strain is ε_1_.
(ii) Cool the sample to room temperature. Five minutes later, unload. The resulted strain is ε_2_.
(iii) Heat to 90 °C for recovery. The final strain is recorded to be ε_3_.

Each sample was tested continuously for three cycles. The shape fixity ratio (R_f_) and shape recovery ratio (R_r_) were calculated by:

(1) $$R_{f}=\frac{\varepsilon_{2}-\varepsilon_{0}}{\varepsilon_{1}-\varepsilon_{0}}\;\%$$

(2) $$R_{r}=(1-\frac{\varepsilon_{3}-\varepsilon_{0}}{\varepsilon_{2}-\varepsilon_{0}})\;\%$$

Ultraviolet–visible (UV–vis) spectra were acquired using a Lambda 950 spectrophotometer (PerkinElmer, Waltham, USA) with a resolution of 1 nm. The polymers were dissolved in CH_2_Cl_2_. After irradiation under a 25 W UV light for a required period of time, they were scanned in the range from 200 to 900 nm.

Uniaxial tensile were carried out at room temperature using an Instron 5567 (Instron Corporation, Norwood, MA, USA). The applied strain rate was 0.17 min^−1^ and the samples were 30 mm × 4 mm × 1.5 mm (length × width × thickness). Both a cyclic uniaxial stretching test to different strains (namely, 10%, 20%, 50% and finally 70%, repeated three times at each prescribed strain) and uniaxial stretching to fracture test were performed.

Herein, the stress and strain are meant for the engineering stress and engineering strain.

Debonding may occur in the normal direction (tension) or tangential direction (shearing). There are some different ways to characterize the bonding strength. To compare the strength of bonding with/without UV cross-linking in a simple way (i.e., via lap joint shear testing. Refer to ASTM D-3164), two pieces of C-UPy-HTPB-UPy-5 wt% film with 0.5 cm in width were overlapped by 2 cm. For the UV bonded sample, the overlapped area was irradiated under a 25 W UV light for 2 h (optimized toughness. Refer to Section 3.2.1). The other piece was heated to 100 °C (well above the temperature that un-cross-linked material is able to flow. Refer to Section 3.3) and then cooled in air to room temperature. Subsequently, the debonding test (shearing) was carried out via uniaxial stretching at a strain rate of 2.8 × 10^−3^/s (gauge length 3 cm).

A piece of cross-linked film with a mass of m_0_ was immersed in CH_2_Cl_2_ for one day for swelling, and then dried in a vacuum-drying oven at room temperature for 12 h. The mass of the dried film was measured to be m_1_. The gel content (G) was calculated by:

(3) $$G=\frac{m_{1}}{m_{0}}\times 100\%$$

## 3. Results and Analysis

### 3.1. Synthesis of C-UPy-HTPB-UPy

UPy-HTPB-UPy is synthesized according to Scheme S1 in the supplementary material. Firstly, UPy-NCO is synthesized following the method reported in [17]. Subsequently, hydroxyl-terminated polybutadiene (HTPB, Mn = 3800–4600 g/mol) reacts with UPy-NCO to form UPy-terminated UPy-HTPB-UPy. The results of ^1^H NMR in Figure S1 confirm the successful synthesis of UPy-HTPB-Upy, which was confirmed by the appearance of the typical peaks at δ (ppm) = 13.11 (CH_3_N*H*), δ = 11.86 (CH_2_NH(CO)N*H*), and δ = 10.19 (CH_2_N*H*(CO)NH) of UPy and the occurrence of the peaks at δ = 5.56–4.96 of olefins.

A prescribed amount of DMPA (photo-initiator) is added in the chloroform solution of UPy-HTPB-UPy to form a homogeneous mixture. After revolution evaporating, a solid piece is resulted due to the hydrogen-bond interaction between UPy groups. After photo irradiation under 356 nm UV, the material is cross-linked via radical reaction of the unsaturated double bonds in HTPB chains to form C-UPy-HTPB-UPy.

#### 3.1.1. Quadrupolar Hydrogen Bond

**Figure 2:**
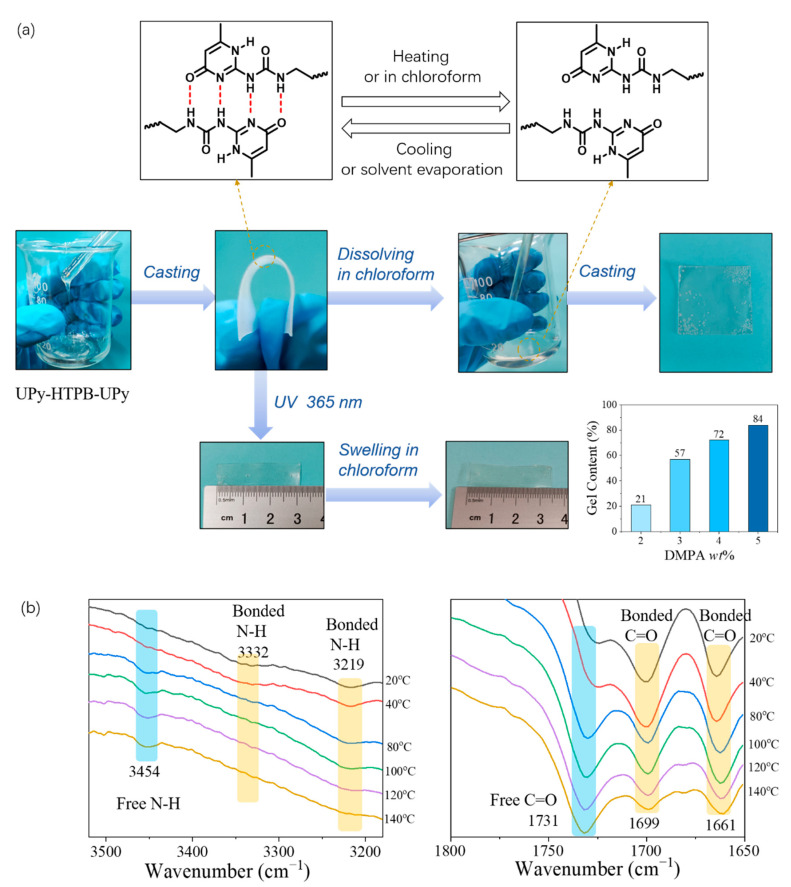
Quadrupolar hydrogen bonding between UPy unites: (**a**) formation and breakdown of hydrogen bond; recasting via hydrogen-bonding interaction, swelling after UV cross-linking and DMPA wt% dependent gel content (%); and (**b**) temperature-dependent (upon heating) FTIR spectra of UPy-HTPB-UPy at N–H and C=O regions.

The quadruple hydrogen bond between polymer chains is the key for the thermo-plasticity and solubility of UPy-HTPB-UPy (refer to Figure 2a). The formation and breakdown of quadruple hydrogen-bond interaction in UPy groups via thermal cycling or solvent (e.g., chloroform) treatment have been well documented in the literature [7,8,17]. As revealed in Figure 2b, un-cross-linked UPy-HTPB-UPy can be dissolved in chloroform. After solvent evaporation, a solid piece, flexible in bending, is obtained. Reshaping can be carried out via repeating this process. Same as most other thermoplastics, for this material, heating to melt the material is an alternative for reshaping. Gel content (in %) is about a linear function of the amount of DMPA in wt%.

The FTIR spectra of C-UPy-HTPB-UPy-*n* wt% and the variable temperature FTIR of UPy-HTPB-UPy-4 wt% are shown in Figure S2 in the supplementary material. FTIR spectra of N–H and C=O at different temperatures upon heating, as shown in Figure 2b, confirms that the quadruple hydrogen bond in UPy groups can be removed. The stretching vibration peak of 3219 cm^−1^ represents hydrogen-bonded N–H in UPy groups. Upon heating from room temperature to 140 °C, the stretching vibration of N–H gradually shifts to 3454 cm^−1^. This proves that the hydrogen-bonding interaction between N–H is eliminated, and becomes free N–H. For C=O, the peaks at 1699 cm^−1^ and 1661 cm^−1^ represent hydrogen-bonded C=O in UPy groups, and their intensity gradually decreases as the temperature increases. On the other hand, the peak of 1731 cm^−1^ represents free C=O in UPy groups, and its intensity gradually increases with the increase in temperature. Hence, upon heating to 80 °C and above, the quadruple hydrogen bonding formed by UPy groups disappears.

#### 3.1.2. UV Cross-Linking

When UPy-HTPB-UPy is irradiated under UV (wavelength: 365 nm), DMPA generates free radicals, which trigger the free radical reaction of the olefin double bond in HTPB chains, forming cross-linking points between molecular chains. The rather unique feature of UPy-HTPB-UPy is that UV cross-linking can be carried out at room temperature, while it is solid. After such a kind of solid-state UV cross-linking, the resulting C-UPy-HTPB-UPy cannot be dissolved, but swelling instead, in chloroform solution (Figure 2b). The gel content (in %) depends on the wt% of DMPA added in.

In the next step, the cross-linking condition of UPy-HTPB-UPy at room temperature under UV illumination was explored.

**Figure 3:**
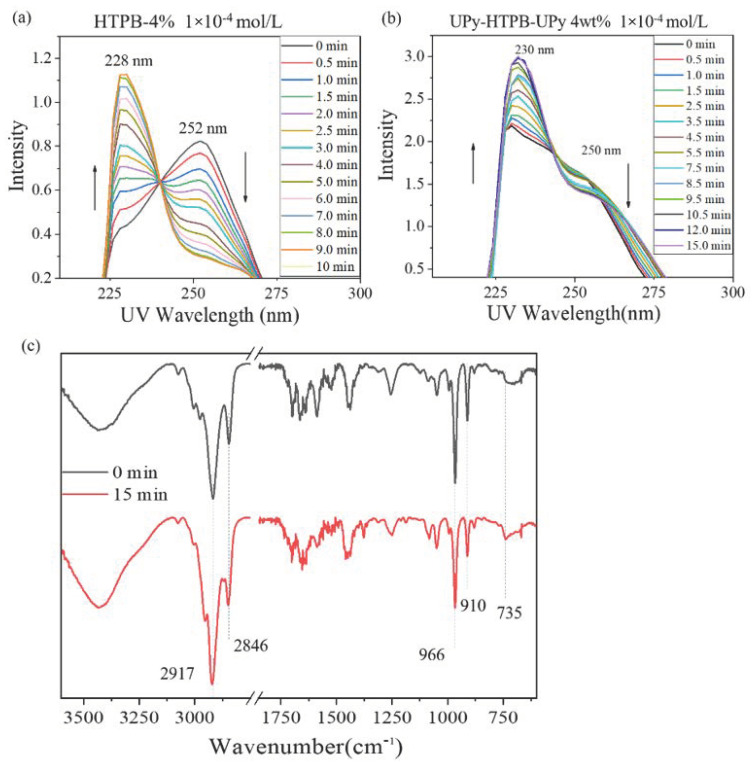
Ultraviolet spectra of (**a**) HTPB-4% and (**b**) UPy-HTPB-UPy-4% with different irradiation times under 365 nm UV; and (**c**) FTIR spectra of UPy-HTPB-UPy-4% before and after UV cross-linking (right: zoomed-in view).

First, the reaction of HTPB under DMPA initiation is studied. HTPB and 4 wt% DMPA are dissolved in chloroform with a concentration of 1 × 10^−4^ mol/L, and then is placed under a 25 W 365 nm UV light. The reaction of double bonds in HTPB chains is induced. The UV spectra in Figure 3a show that the 252 nm peak of C=C bond in HTPB disappears and a new peak appears at 228 nm, which becomes more significant with the increase in the illumination time. It appears that the reaction can be finished in 10 min.

In the next step, the reaction of UPy-functionalized HTPB is investigated. A chloroform solution of UPy-HTPB-UPy and 4 wt% DMPA with a concentration of 1 × 10^−4^ mol/L are used. It should be noted that the initial peak intensity of the C=C bond is not as strong as that of the pure HTPB. As compared with the reaction process of purity HTPB, some double bonds should have already reacted during the preparation process. Figure 3b reveals the same trend, and the C=C reaction finishes in approximately 12 min since there is not much difference between the curves of 12 min and 15 min. According to the FTIR spectra in Figure 3c, the peaks at 2917 cm^−1^ and 2846 cm^−1^ are associated with the stretching vibration of –CH- and –CH_2_- groups, the peak at 735 cm^−1^ is due to cis-1,4 isomers, and the peaks at 910 cm^−1^ and 966 cm^−1^ are signed to the traces of 1,2-vinyl and trans-1,4 isomers, respectively. [19,20,21] The peak intensity of ethylenic bond stretching is apparently weakened when compared with that of alkane bond, which indicates that the cross-linking reaction has occurred.

From the real engineering application point of view, in order to shorten the reaction time, a high intensity of UV light (e.g., high-pressure mercury lamp) may be used.

Typical DSC results in Figure S3 in the supplementary material reveal that there are some slight differences after cross-linking. In particular, the trough in the first heating process turns out to be wider after cross-linking. In the second heating process, the magnitude of the trough in the sample without cross-linking becomes less significant, while there is no more apparent trough in the cross-linked piece. It is noticed that there is not any peak upon cooling in both samples. Cross-linking affects the chain arrangement and turns polymers to be more vitrified [19].

### 3.2. Mechanical Properties

#### 3.2.1. Effect of Photo-Initiator Content and Curing Time

As shown in Figure S4a in the supplementary material, i.e., the stress versus strain curve of UPy-functionalized HTPB in cyclic uniaxial tension, UPy-HTPB-UPy has a relatively lower fracture strain (less than 30%). This is similar to the cross-linked polybutadiene reported in [22], in which the quadruple hydrogen-bonding interactions of UPy play the same role as chemical cross-linking in the polybutadiene chains. When compared with other UPy-functionalized polymers, [9,11] it appears that the relatively lower strain observed here is mostly due to the structure of HTPB itself.

**Figure 4:**
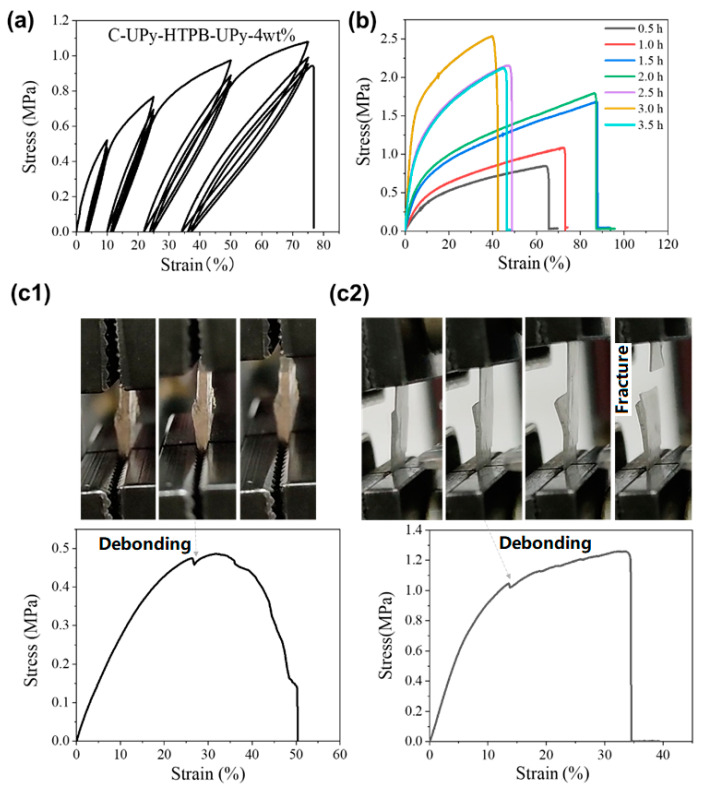
(**a**) Stress versus strain relationship of C-UPy-HTPB-UPy-4 wt% in cyclic uniaxial tension to 10%, 20%, 50%, and finally 70% strain (three cycles at each prescribed strain) (cross-linking time: 2 h); (**b**) uniaxial stretching to fracture of UV cross-linked UPy-HTPB-Upy-4 wt% for different UV cross-linking times; and (**c**) (**top**) snapshot of debonding experiment (via stretching, from left to right) and (**bottom**) stress–strain curve of two UPy-HTPB-UPy strips bonded by (**c1**) quadruple hydrogen bonding and (**c2**) after UV cross-linking, respectively.

Figure 4a is the stress versus strain relationship of C-UPy-HTPB-UPy-4 wt% in cyclic uniaxial tension to the 10%, 20%, 50%, and finally 70% strain (three cycles at each prescribed strain). Here, refer to Figure S4b–d in the supplementary material for typical results of other cross-linked samples with different amounts of photo-initiator. Table S1 in the supplementary material summarizes the average and standard deviation of the fracture stress and fracture strain of all C-UPy-HTPB-UPy-*n* wt%. It is apparent that after UV cross-linking, the mechanical property of the material can be improved. As we see, the fracture strain doubles from 25% for UPy-HTPB-UPy to 56.7% for C-UPy-HTPB-UPy-2 wt%. With the increase in DMPA, the fracture stress increases continuously, while the fracture strain reaches the maximum in C-UPy-HTPB-UPy-4 wt%. Hence, C-UPy-HTPB-UPy-4 wt% appears to be the best. Together with Figure 4b, in which the cross-linking time is varied from 0.5 h to 3.5 h, we can conclude that both the amount of photo-initiator and UV cross-linking time affect the mechanical property of the resulted materials. For the same UV cross-linking time (Figure 4a and Figure S4b–d and Table S1 in the supplementary material), 4 wt% of photo-initiator achieves the best performance over 1.0 MPa of ultimate stress and 78% fracture strain. For the same amount of photo-initiator (4 wt%), while varying the cross-linking time from 0.5 h to 3.5 h (refer to Figure 4b), the optimal UV cross-linking time is identified to be approximately 1.5 to 2 h for both high fracture stress and high fracture strain. Further extension of the cross-linking time enhances the fracture stress at the cost of a lower fracture strain, i.e., the material is further hardened but less ductile.

#### 3.2.2. Bonding

A strong bonding between layers is required in 3D printing. For the materials developed here, quadruple hydrogen bonding is formed in the printing process. UV cross-linking is expected to further strengthen the bonding. In Figure 4(c1,c2), the bonded samples with/without UV cross-linking are stretched to fracture. As we can see, without cross-linking, the failure is due to debonding, while the cross-linked piece fails due to the fracture of the material. Although partial debonding is observed in the cross-linked sample as well, the debonding start stress is approximately 1 MPa, which is well above the debonding start stress of the un-cross-linked sample (approximately 0.46 MPa). Without cross-linking, the maximum pulling stress is approximately the same as the debonding start stress (0.46 MPa, hydrogen bonding only), which is lower than the fracture stress of UPy-HTPB-UPy (refer to Figure S4 in the supplementary material). After cross-linking, the maximum pulling stress is dramatically increased to approximately 1.2 MPa, which is the fracture stress of C-UPy-HTPB-UPy.

### 3.3. DMA and Shape Memory Behavior of C-UPy-HTPB-UPy

**Figure 5:**
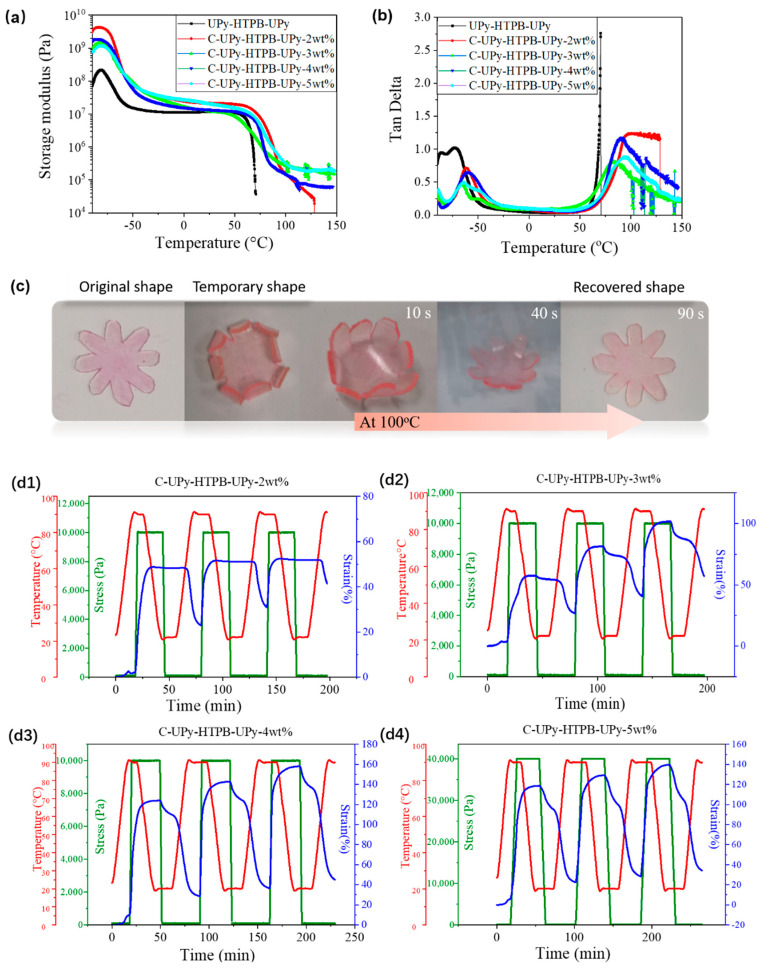
DMA results of (**a**) storage modulus and (**b**) tangent delta. (**c**) Shape memory effect of C-UPy-HTPB-UPy-4 wt% (rhodamine staining to color the material into red) and (**d1**–**d4**) three shape memory cycles of C-UPy-HTPB-UPy-*n* wt%, *n* = 2, 3, 4 and 5 (via DMA).

DMA results in Figure 5a,b (also refer to Figure S5 in the supplementary material) reveal that the storage modulus of all materials (with/without UV cross-linking) rapidly drops in two temperature ranges upon heating from −90 °C to 150 °C, at approximately −70 °C (due to glass transition), and then at approximately 80 °C (due to the debonding of the quadruple hydrogen bond of UPy). Upon heating to approximately 80 °C, UPy-HTPB-UPy does not have the capability to maintain the shape upon loading, while all C-UPy-HTPB-UPy-*n* wt% (UV cross-linked) only soften further. Features (peak or dramatic increase) correspond to the glass transition (peak), and debonding (dramatic increase or peak) can be observed in their Tan delta curves as well. Hence, based on the quadruple hydrogen bond of UPy which is reversible upon thermal cycling, the heating-responsive shape memory effect is expected in C-UPy-HTPB-UPy.

Recall the DSC result of the un-cross-linked sample in Figure S3b in Supplementary. Apparently, this type of room-temperature UV cross-linkable vitrimer is different from that reported in [6], which melts upon heating to approximately 65 °C (based on DSC) and then the reversible cross-linking starts to gradually disappear from approximately 80 °C (according to DMA). Only upon heating to approximately 105 °C does it becomes easy to flow (according to DMA).

In Figure 5c, a piece of flower-shaped C-UPy-HTPB-UPy-4 wt% (1.5 mm thick) is programmed via folding at 100 °C. Upon heating to 100 °C again, it gradually recovers its original shape in 90 s.

More DMA results (three shape memory cycles in each test) are presented in Figure 5d. Refer to Table S2 in the supplementary material for the shape fixity ratio and shape recovery ratio of all cross-linked samples in shape memory cycling and refer to Equations (1) and (2) for how the shape fixity ratio and shape recovery ratio are worked out. Normally, it is not possible to simultaneously achieve high shape fixity ratio and high shape recovery ratio in shape-memory polymers [5]. As we can see from Table S1 in the supplementary material, from the second cycle, C-UPy-HTPB-UPy-4 wt% appears to be the best in both shape fixed ratio and shape recovery ratio.

Without UV cross-linking, the quadruple hydrogen bond of UPy, which is reversible upon thermal cycling, indicates that UPy-HTPB-UPy is essentially a vitrimer-like polymer. However, slightly different from that reported in [6], UPy-HTPB-UPy can be cross-linked at room temperature, and it becomes easy to flow upon heating to the temperature that the quadruple hydrogen bond disappears (refer to Figure S3 in the supplementary material and Figure 5a).

**Figure 6:**
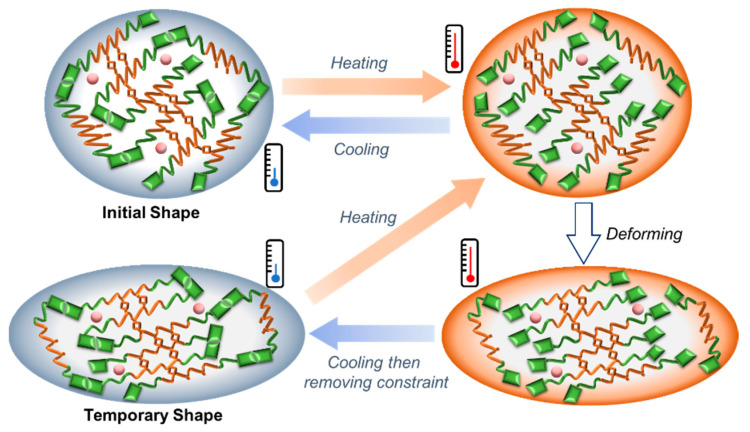
Shape memory mechanism of C-UPy-HTPB-UPy.

After cross-linking, C-UPy-HTPB-UPy has the heating-responsive SME, in which the UPy group serves as the switch. The underlying mechanism for the shape memory effect is illustrated in Figure 6. Upon heating to the temperature that the quadruple hydrogen bond is removed, C-UPy-HTPB-UPy can be deformed easily. After cooling back to room temperature, the quadruple hydrogen bond is re-established, and the programmed (temporary) shape mostly remains after the constraint is removed. Being heated once again to above the temperature that the quadruple hydrogen bond is removed, C-UPy-HTPB-UPy recovers its initial shape.

## 4. Potential Applications in Additive Manufacturing

**Figure 7:**
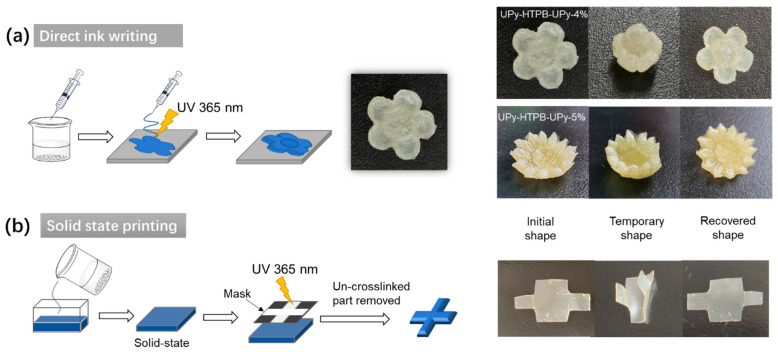
Applications in additive manufacturing: (**a**) DIW and (**b**) solid-state VAM.

The room-temperature UV cross-linkable UPy-HTPB-UPy (pre-loaded with DMPA) developed here has great potential in additive manufacturing. Before cross-linking, this material is vitrimer-like. Hence, it can be 3D printed via FDM. The room-temperature UV cross-linkable feature (either during or after FDM printing) enables not only stronger bonding, but also the whole printed piece to be strengthened. As UPy-HTPB-UPy is dissolvable in chloroform, direct ink writing (DIW) is also applicable. As illustrated in Figure 7a, during DIW, UV exposure enhances the strength between layers. Two printed flowers, one is UPy-HTPB-UPy-4 wt% and the other is UPy-HTPB-UPy-5 wt%, have good heating-responsive shape memory effect (following the same procedure as reported in Figure 5c).

Recently, rapid VAM in solid state has been demonstrated using a special thermal gel [4]. For a hard polymeric version, UV cross-linkable vitrimer has been suggested as the material [6]. The material developed here is just right for proof-of-the-concept. As illustrated in Figure 7b, after heating to melt UPy-HTPB-UPy (pre-loaded with DMPA), the liquid material is poured into a container. The material becomes solid when it is back to room temperature. Subsequently, UV cross-linking through a mask results in the cross-shaped area becoming thermoset. The un-cross-linked part can be removed either upon heating or via immersing in the right solvent. The remaining, in a cross shape, has good heating-responsive shape memory effect, which ensures the maintenance of the precise dimensions of the printed piece after the separation process. Here, refer to Movie S1 in the supplementary material for a demonstration of the process. In the video, the separation of the cross-linked cross shape from the un-cross-linked part is carried out manually after pre-heating to soften the sample. As a matter of fact, this is more in favor in real engineering applications. A more sophisticated UV cross-linking system, e.g., such as that used in [2], can be used to realize actual rapid AM in solid state in any environment at any time [3].

## 5. Conclusions

A new UV cross-linkable vitrimer-like polymer UPy-HTPB-UPy with two types of functional groups is reported here. One functional group is the UPy group with quadruple hydrogen bond, which is reversible upon thermal cycling and dissolvable in the right solvent. The other functional group is the double bond in the HTPB-chain segment, which is UV cross-linkable (after mixing with the photo-initiator) at room temperature in the solid state. The density of cross-linking can be tailored. Before cross-linking, the material is vitrimer-like while, after cross-linking, the material has the shape memory effect.

Some potential applications of this UV cross-linkable vitrimer-like material in additive manufacturing are demonstrated. In the cases of FDM and DIW, UV cross-linking enhances not only the bonding between layers, but also the material itself. Furthermore, the concept of rapid VAM in the solid state is confirmed using this UV cross-linkable vitrimer-like material, which demonstrates the possibility for rapid AM anytime and anywhere.

## Supplementary Materials

The following supporting information can be downloaded at: https://www.mdpi.com/article/10.3390/polym14112203/s1, Scheme S1: Synthetic route of UPy-HTPB-UPy. Figure S1: ^1^H NMR of (a) UPy-NCO, (b) HTPB, and (c) UPy-HTPB-UPy confirms that UPy unites are connected to HTPB; Figure S2: (a) FTIR spectra of C-UPy-HTPB-UPy-*n* wt% films with different weight content of photo-initiator (*n* = 0%, 2% 3%, 4% and 5%), (b) FTIR spectra of UPy-HTPB-UPy-4 wt% at different temperatures; Figure S3: DSC results of (a) C-UPy-HTPB-UPy-5 wt% and (b) UPy-HTPB-UPy; Figure S4: cyclic uniaxial tension of (a) UPy-HTPB-UPy and (b–e) C-UPy-HTPB-UPy-*n* wt% to 10%, 20%, 50%, and finally 70% strain (three cycles at each prescribed strain) (all samples were cross-linked under 25 W 365 nm UV light for 2 h); Figure S5: DMA results of (a)~(d) C-UPy-HTPB-UPy-nwt% (*n* = 2, 3, 4, 5) and (e) UPy-HTPB-UPy; Table S1: uniaxial tension to fracture of C-UPy-HTPB-UPy-*n*%; Table S2: shape fixation ratio (Rf) and shape recovery ratio (Rr) of C-UPy-HTPB-UPy-*n*% in three shape memory cycles carried out by DMA; Movie S1: demonstration of UV cross-linking in solid state at room temperature of vitrimer.
