# Supplementary material for: Room-Temperature Solid-State UV Cross-Linkable Vitrimer-like Polymers for Additive Manufacturing
Source: Polymers (Basel). 2022 May 29;14(11):2203. doi: 10.3390/polym14112203 (PMC9182850; doi:10.3390/polym14112203)
Supplement: Supplementary file 1 [file polymers-14-02203-s001.zip › polymers-1707355-supplementary.pdf]

Supplementary

Room temperature solid-state UV cross-linkable vitrimer-like polymers for additive manufacturing

Jian Chen[†], Ya Wen[†], Lingyi Zeng[†], Xinchun Wang[†], Hongmei Chen*[,†], Wei Min Huang*[,‡], Yuefeng Bai[†], Wenhao Yu[†], Keqing Zhao[†], and Ping Hu*[,†]

[†] College of Chemistry and Materials Science, Sichuan Normal University, Chengdu 610066, China.

[‡] School of Mechanical and Aerospace Engineering, Nanyang Technological University, Singapore 639798, Singapore.

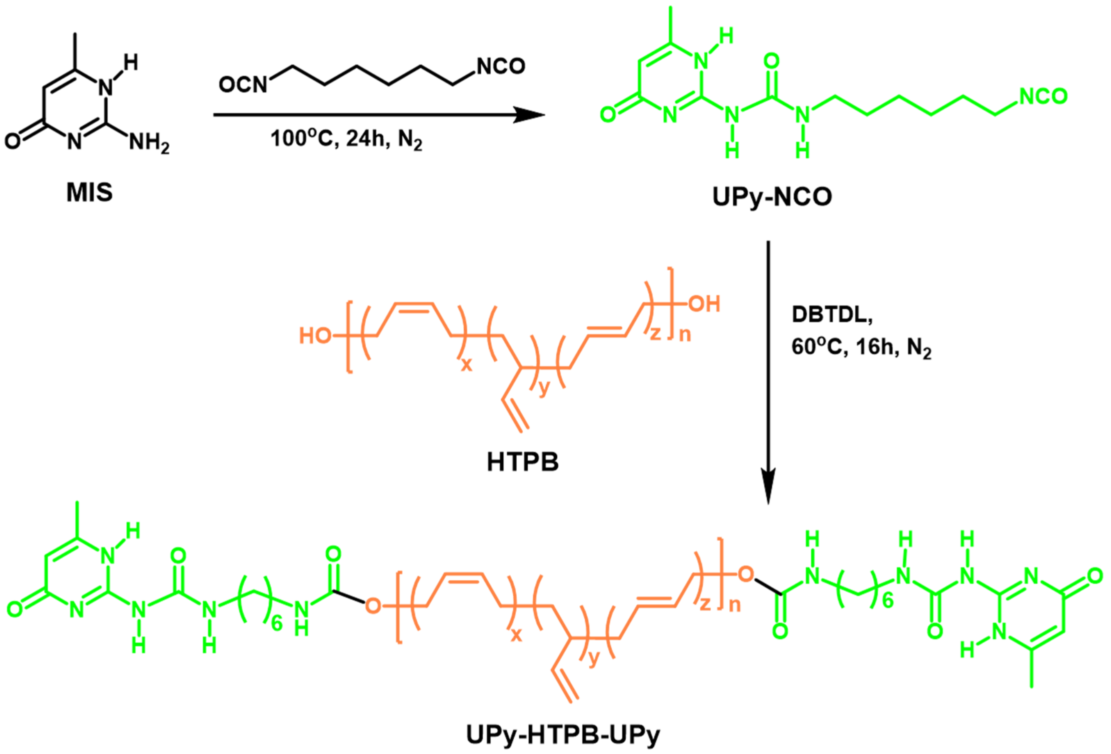

Scheme S1. Synthetic route of UPy-HTPB-UPy.

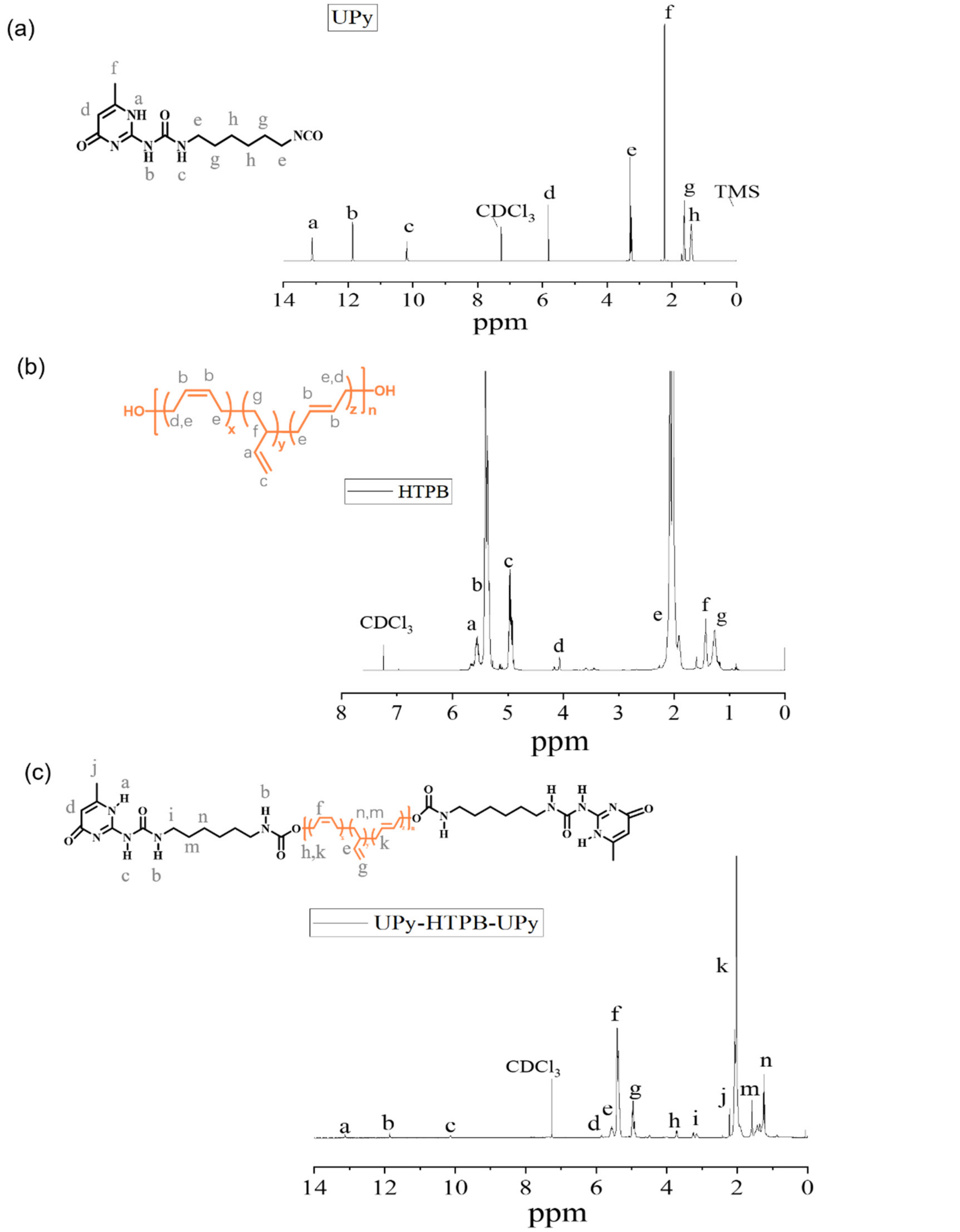

Figure S1. ^{1}H NMR results of (a) UPy-NCO, (b) HTPB, and (c) UPy-HTPB-UPy confirm that UPy unites are connected to HTPB.

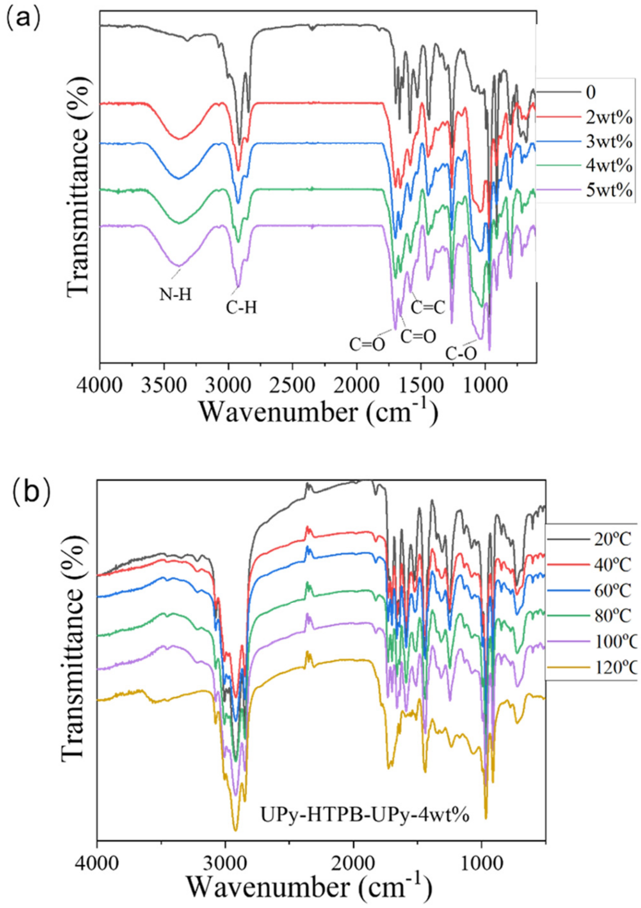

Figure S2. (a) FTIR spectra of C-UPy-HTPB-UPy-*n*wt% films with different weight content of photo initiator (*n* = 0%, 2% 3%, 4% and 5%); (b) FTIR spectra of UPy-HTPB-UPy-4wt% at different temperatures.

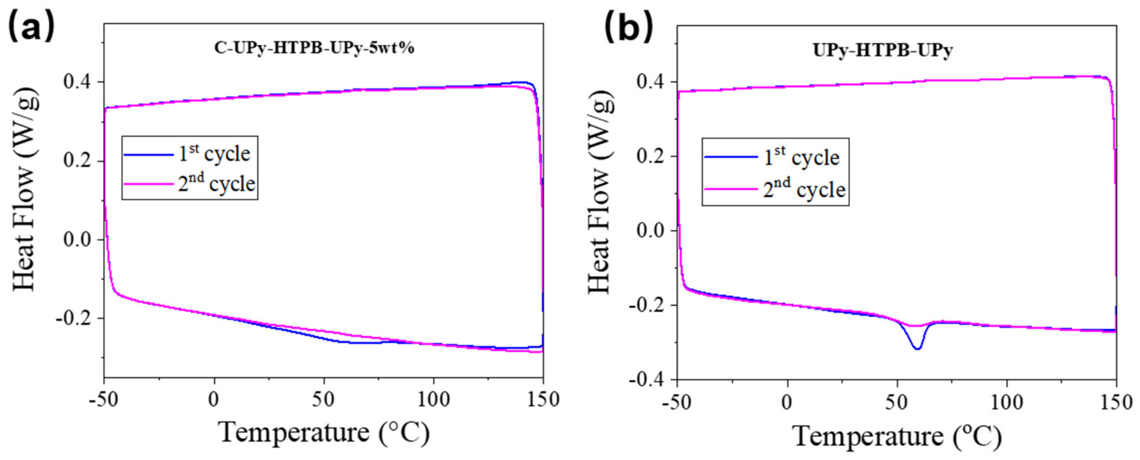

Figure S3. DSC results of (a) C-UPy-HTPB-UPy-5wt% and (b) UPy-HTPB-UPy.

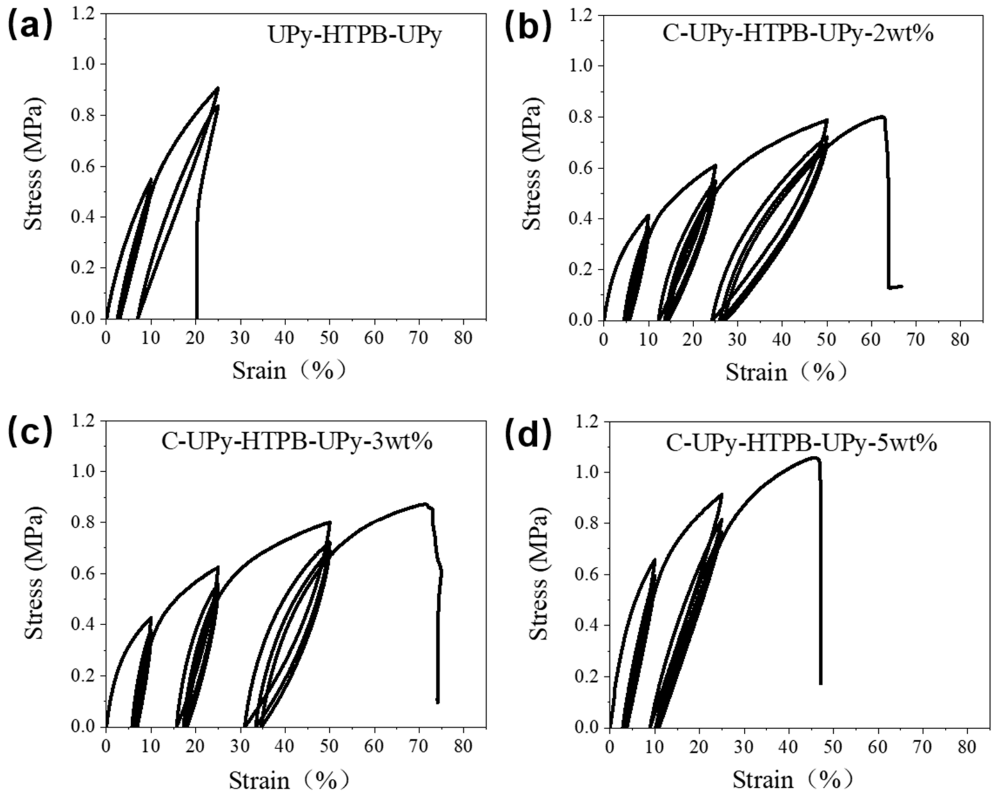

Figure S4. Cyclic uniaxial tension results of (a) UPy-HTPB-UPy and (b)-(e) C-UPy-HTPB-UPy-*n*wt% to 10%, 20%, 50%, and finally 70% strain (three cycles at each prescribed strain) (all samples were cross-linked under 25 W 365 nm UV light for 2 h).

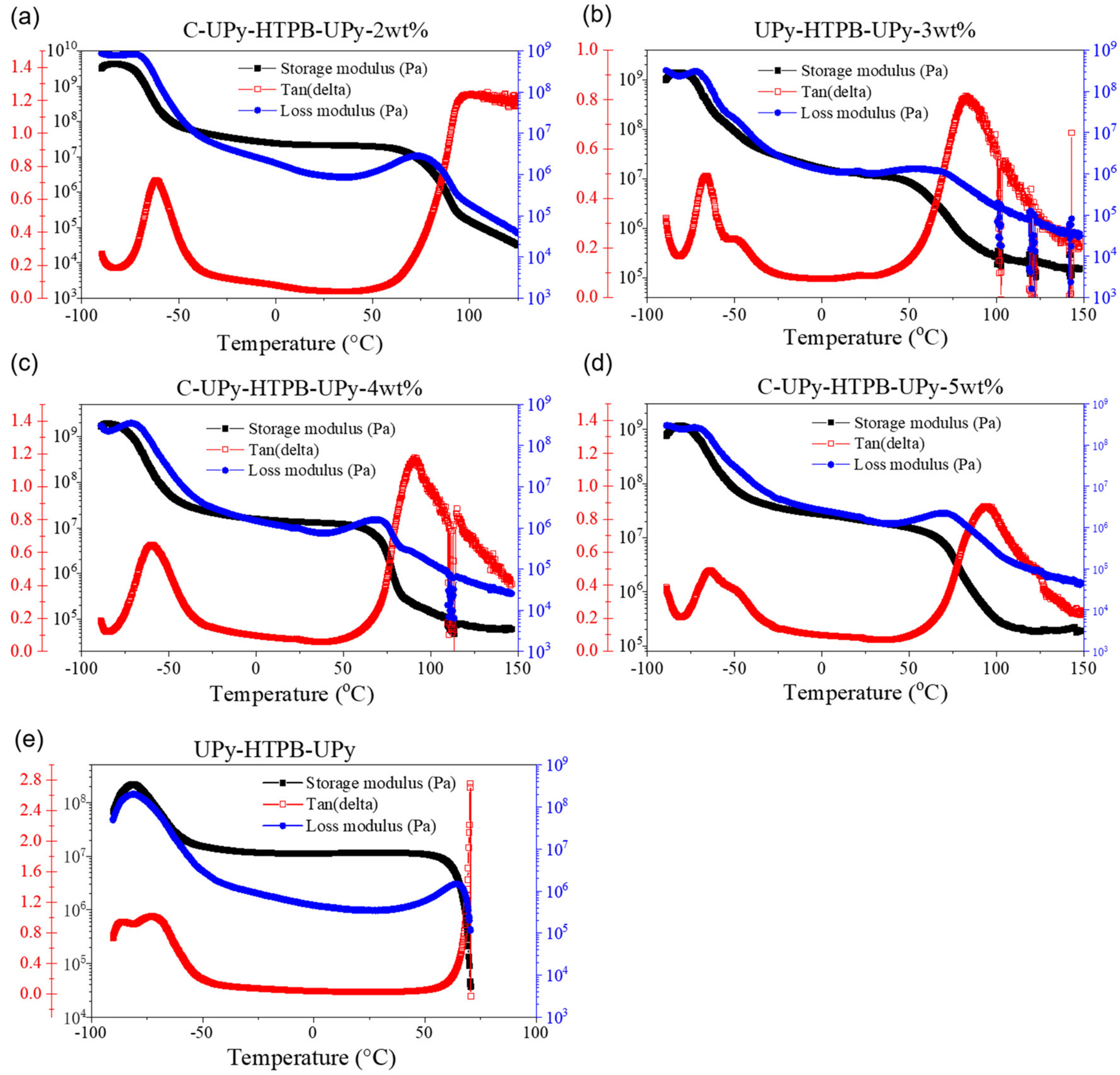

Figure S5. DMA results of (a)~(d) C-UPy-HTPB-UPy-*n*wt% (*n*=2,3,4,5) and (e) UPy-HTPB-UPy.

Table S1. Uniaxial tension to fracture of C-UPy-HTPB-UPy-*n*%.

n	2	3	4	5
Fracture stress σ（MPa）	0.84±0.05	0.98±0.09	1.05±0.02	1.15±0.02
Fracture strain ε（%）	56.7±4.2	67.9±2.7	78.1±6.96	50.1±3.0

Table S2. Shape fixation ratio (R_f) and shape recovery ratio (R_r) of C-UPy-HTPB-UPy-*n*% in three shape memory cycles carried out by DMA.

n	2wt%			3wt%			4wt%			5wt%		
	1st	2nd	3rd	1st	2nd	3rd	1st	2nd	3rd	1st	2nd	3rd
R_f (%)	99	99	99	95	87	77	89	83	82	81	73	82
R_r (%)	52	71	50	50	70	65	74	92	92	76	92	94

Movie S1. Demonstration of UV cross-linking in solid state at room temperature.
https://www.youtube.com/watch?v=0r3455sb8Lg
